# FIZZ1 promotes airway remodeling through the PI3K/Akt signaling pathway in asthma

**DOI:** 10.3892/etm.2014.1580

**Published:** 2014-02-24

**Authors:** JUNFEI WANG, FEI LI, MENGMENG YANG, JINXIANG WU, JIPING ZHAO, WENBIN GONG, WEN LIU, WENXIANG BI, LIANG DONG

**Affiliations:** 1 Department of Pulmonary Medicine, Qilu Hospital of Shandong University, Jinan, Shandong 250012, P.R. China; 2 Department of Pulmonary Medicine, People’s Hospital of Rizhao, Rizhao, Shandong 276800, P.R. China; 3 Department of Pulmonary Medicine, The Second Hospital of Shandong University, Jinan, Shandong 250100, P.R. China; 4 Institute of Biochemistry and Molecular Biology, School of Medicine, Shandong University, Jinan, Shandong 250012, P.R. China

**Keywords:** asthma, airway remodeling, found in inflammatory zone 1, epithelial-mesenchymal transition, phosphoinositide 3-kinase/protein kinase B signaling pathway

## Abstract

Found in inflammatory zone 1 (FIZZ1) plays a vital role in pulmonary inflammation and angiogenesis. In addition, FIZZ1 plays a role in the early stages of airway remodeling in asthma by increasing the expression of α smooth muscle actin (α-SMA) and type I collagen. However, the role of FIZZ1 in the airway remodeling of asthma remains unclear. In the present study, FIZZ1 was identified to be upregulated in ovalbumin (OVA)-induced asthmatic mice, along with phosphorylated protein kinase B (Akt). Following FIZZ1 recombinant protein co-culture in the murine lung epithelial-cell line, Akt phosphorylation was upregulated, however, following transfection with FIZZ1-small hairpin RNA, the phosphorylation levels were decreased. The variation in α-SMA and type I collagen expression levels was consistent with the Akt phosphorylation levels. Intratracheal administration of LY294002 and Akt inhibitor IV to the asthmatic mice was capable of reducing airway inflammation, downregulating the expression of α-SMA, type I collagen and fibronectin-1 and increasing the expression of E-cadherin. In conclusion, the present study demonstrated that FIZZ1 promoted airway remodeling in asthma via the phosphoinositide 3-kinase (PI3K)/Akt signaling pathway. Blocking the PI3K/Akt signaling pathway may attenuate the early stages of airway remodeling induced by OVA by regulating the abnormal process of epithelial-mesenchymal transition.

## Introduction

Asthma is a chronic inflammatory disease of the bronchial airway characterized by chronic airway inflammation, airway hyperresponsiveness, mucus overproduction and airway remodeling (1,2). A previous study demonstrated that airway remodeling is a characteristic feature of asthma and may be observed from the early stages (3). Structural and functional changes to the epithelial cells are significant in the process of airway remodeling (3,4) and airway epithelial cells are able to promote the process through epithelial-mesenchymal transition (EMT) (5). During the process of EMT, the expression levels of type I collagen, fibronectin-1 and α-smooth muscle actin (α-SMA) have been observed to be upregulated, while the expression of the epithelial marker, E-cadherin, has been shown to be reduced (6). The abnormal process of EMT was observed as a central pathological and physiological characteristic in the early stages of airway remodeling (7).

Found in inflammatory zone 1 (FIZZ1) was first reported in 2000 by Holcomb *et al* in allergic pulmonary inflammation (8) and was shown to play a vital role in pulmonary inflammation and angiogenesis (9). Our previous study demonstrated that FIZZ1 was vital in airway remodeling in asthma and was capable of increasing the expression levels of α-SMA and type I collagen in the early stages of airway remodeling (10).

However, the mechanism by which FIZZ1 functions in the process of airway remodeling remains unclear. In the present study, the hypothesis that FIZZ1 may activate the phosphoinositide 3-kinase (PI3K)/protein kinase B (Akt) signaling pathway through promoting Akt phosphorylation *in vitro* was investigated. In addition, the effect that blocking the PI3K/Akt pathway has on decreasing inflammatory cell infiltration and alleviating airway remodeling via regulating the process of EMT was investigated.

## Materials and methods

### Animals

Specific-pathogen-free, female BALB/c mice (age, 8–10 weeks; weight, 20±2 g; Animal Experiment Center of Shandong University, Shandong, China) were sensitized on days 1 and 14 by intraperitoneal injection of 20 μg ovalbumin (OVA; Sigma-Aldrich, St. Louis, MO, USA) and 4 mg Al(OH)_3_ (Sigma-Aldrich) suspended in 0.2 ml saline. On days 21–23 following the initial sensitization, the mice were challenged for 30 min with an aerosol of 1% (wt/vol) OVA in saline using an ultrasonic nebulizer (PARI BOY SX, Starnberg, Germany), while saline alone was used to challenge the control group. LY294002 (7.5 mg/kg body weight; Cell Signaling Technology, Inc., Beverly, MA, USA), Akt inhibitor IV (5 mg/kg body weight; Santa Cruz Biotechnology, Inc., Santa Cruz, CA, USA) or saline were administered intratracheally 2 h prior to each OVA aerosol challenge (Table I). All the animal experiments were approved by the Institutional Animal Care and Use Committee of Shandong University (Jinan, China).

### Analysis of airway responsiveness

At 24 h after the last challenge, the mice were anesthetized by intraperitoneal injection of chloral hydrate (4 mg/kg body weight). Methacholine was administered at a concentration of 0, 4, 8, 12 or 16 g/l. Measurements of airway hyperresponsiveness were conducted using an animal pulmonary instrument (flexiVent, Hong Kong, China) 1 min after each dose with 2 min between doses. The results were expressed as the maximum resistance following each dose minus the baseline (saline alone) resistance.

### Histological analysis

Lung tissues were fixed in 10% neutral formalin, paraffin-embedded, cut into 4-μm sections and stained with hematoxylin and eosin for examination of inflammatory cell infiltration.

### Immunohistochemistry analysis

Sections were dewaxed, rehydrated and antigen retrieval was performed with 10 mM sodium citrate (pH 6.1). Next, the sections were blocked with 5% bovine serum albumin for 20 mins at 37°C. The sections were incubated with anti-FIZZ1 (1:300), anti-type I collagen (1:300), anti-E-cadherin (l:300) or anti-fibronectin-1 (l:300) antibodies (all Santa Cruz Biotechnology, Inc.) overnight at 4°C. The sections were subsequently incubated with polyclonal goat anti-rabbit immunoglobulins/horseradish peroxidase (1:200) for 30 min at 37°C. The nuclei were counterstained with hematoxylin.

### Murine lung epithelial-12 (MLE-12) cell culture

The MLE-12 cell line was purchased from a cell bank (American Type Culture Collection, Manassas, VA, USA) and cultured in Dulbecco’s modified Eagle’s medium/F12 complete medium with 10% fetal bovine serum, 2 mM glutamine, 100 U/ml penicillin and 100 μg/ml streptomycin at 37°C and 5% CO_2_. Following a 48-h culture, the cells were seeded in 6-well culture plates.

### FIZZ1 recombinant protein co-culture and FIZZ1 small hairpin RNA (shRNA) transfection

The MEL-12 cell line was cultured with FIZZ1 recombinant protein (1 μg/ml; Santa Cruz Biotechnology, Inc.), while the control group used phosphate-buffered saline instead. Subsequent to 24 h, the protein was extracted from the cell line for analysis of the Akt phosphorylation levels and the expression levels of α-SMA and type I collagen.

*Escherichia coli* JM109 bacteria of the FIZZ1-shRNA plasmid (sc-39724-SH; Santa Cruz Biotechnology, Inc.) were amplified in liquid medium. Plasmid DNA was extracted with the Omega D6950 plasmid extraction kit (Omega Bio-tek, Inc., Norcross, GA, USA).

Lipofectamine 2000 reagent (Invitrogen Life Technologies, Carlsbad, CA, USA) was used to transfect the FIZZ1-shRNA plasmid to the cell line, according to the manufacturer’s instructions. After 48 h of incubation, the cells were harvested for analysis of FIZZ1, p-Akt, α-SMA and type I collagen.

An empty plasmid was added to the control and was cultured for the same amount of time. The cells were then collected to analyze the expression of FIZZ1, p-Akt, α-SMA and type I collagen by western blot analysis.

### Quantitative polymerase chain reaction (PCR)

Total RNA was extracted from the mouse lungs with TRIzol reagent (Invitrogen Life Technologies), according to the manufacturer’s instructions, and was reverse transcribed with a PrimeScript 1st Strand cDNA Synthesis kit (Takara Bio, Inc., Shiga, Japan). cDNA was amplified by SYBR Premix *Ex Taq*II (Perfect Real Time; Takara Bio, Inc.) in Roche Light Cycler 2.0. The primers used were as follows: α-SMA forward, 5′-CCACCGCAAATGCTTCTAAGT-3′ and reverse, 3′-GGCAGGAATGATTTGGAAAGG-5′; type I collagen forward, 5′-CGCCATCAAGGTCTACTGC-3′ and reverse, 3′-GAATCCATCGGTCATGCTCT-5′; and GADPH forward, 5′-GTGGCAAAGTGGAGATTGTT-3′ and reverse, 3′-CTCGCTCCTGGAAGATGG-5′. The PCR conditions were 95°C for 10 sec, then 40 cycles at 95°C for 5 sec, 57°C for 30 sec and 72°C for 30 sec. The expression levels of α-SMA and type I collagen were assessed using the comparative Ct method.

### Western blot analysis

In total, 30 μg protein extracted from the lung was separated by 10% SDS-PAGE, transferred onto a polyvinylidene fluoride membrane and subjected to immunoblotting with anti-α-SMA, anti-type I collagen or anti-β-actin antibodies (Santa Cruz Biotechnology, Inc.).

### Statistical analysis

Data are presented as the mean ± SEM. Continuous variables were analyzed using the Student’s t-test between the groups studied. P<0.05 was considered to indicate a statistically significant difference.

## Results

### Asthma model in mice

**Figure 1:**
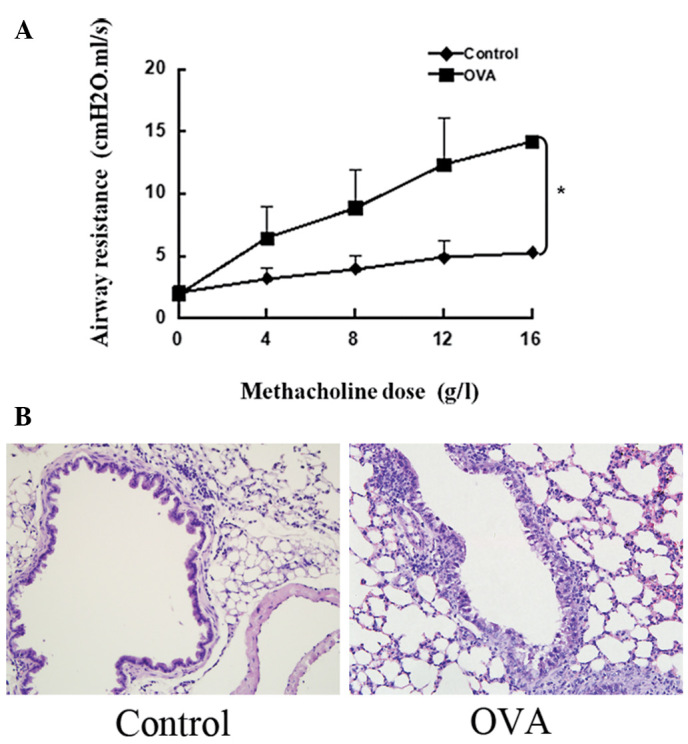
Airway hyperresponsiveness and inflammatory cell infiltration around the airway. (A) Airway hyperresponsiveness developed in the OVA group (^*^P<0.05, vs. saline control). (B) Massive infiltration of inflammatory cells may be observed in the OVA group (hematoxylin and eosin; magnification, ×200). OVA, ovalbumin.

The murine asthma model was confirmed by detecting airway hyperresponsiveness and histopathology. As shown in Fig. 1A, the OVA-treated mice developed airway hyperresponsiveness to inhaled methacholine compared with the saline-challenged group. Furthermore, the OVA aerosol challenge induced the infiltration of inflammatory cells around the airway (Fig. 1B).

### FIZZ1 and p-Akt expression in mice

**Figure 2:**
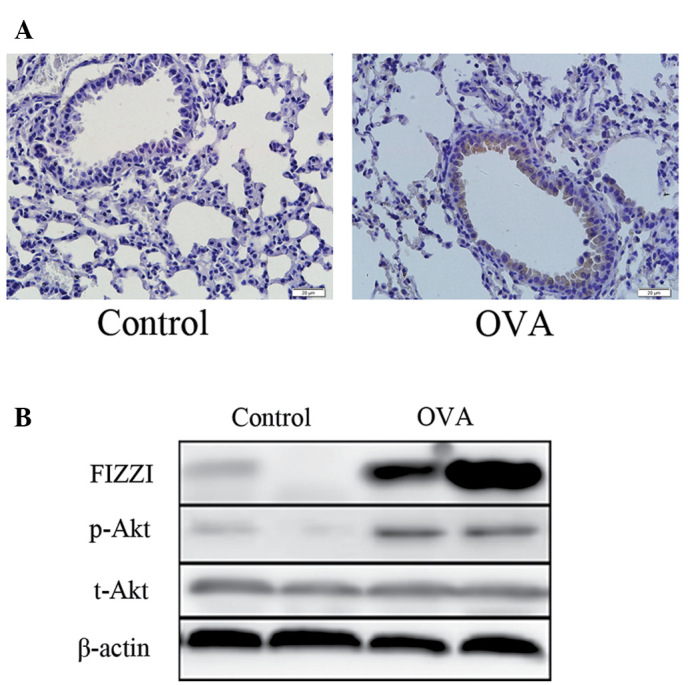
FIZZ1 expression and phosphorylation levels of Akt in the lung tissues. (A) Expression of FIZZ1 was upregulated in the epithelial cells of the airway in the OVA group, as shown by immunohistochemistry (hematoxylin and eosin; magnification, ×400). (B) The same result was demonstrated by western blot analysis and Akt phosphorylation levels also increased in the OVA group. FIZZ1, found in inflammatory zone 1; OVA, ovalbumin.

The expression of FIZZ1 in mouse airway epithelium cells was upregulated in the OVA-induced asthmatic mice compared with the saline control group (Fig. 2A). In terms of protein levels, an enhancement of FIZZ1 expression and Akt phosphorylation was detected in the OVA group by western blot analysis (Fig. 2B).

### Expression of FIZZ1 and Akt phosphorylation in MLE-12 cells following FIZZ1 shRNA transfection by western blot analysis

**Figure 3:**
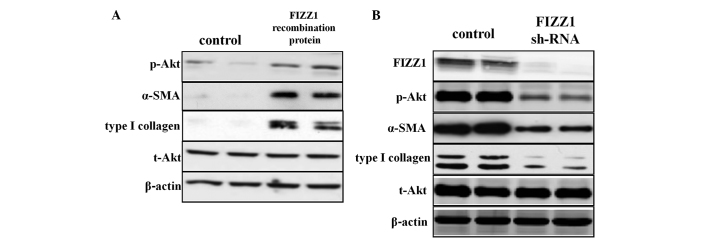
Effect of FIZZ1 on Akt phosphorylation and airway remodeling. (A) Following co-culturing with FIZZ1 recombinant protein, the phosphorylation of Akt was enhanced, as well as α-SMA and type I collagen expression. (B) Upon silencing the expression of FIZZ1, the Akt phosphorylation levels were suppressed. The variation of α-SMA and type I collagen expression was the same as for p-Akt. FIZZ1, found in inflammatory zone 1; Akt, protein kinase B.; α-SMA, α smooth muscle actin.

To verify whether FIZZ1 was able to promote Akt phosphorylation, the MLE-12 cell line was cultured with FIZZ1 recombinant protein following FIZZ1 shRNA transfection and the phosphorylation levels of Akt were detected. Following FIZZ1 recombinant protein co-culture, Akt phosphorylation and α-SMA and type I collagen expression were upregulated (Fig. 3A). The level of FIZZ1 expression in the MLE-12 cells following FIZZ1 shRNA transfection was downregulated, as were the levels of Akt phosphorylation and α-SMA and type I collagen expression (Fig. 3B).

### Effects of LY294002 and Akt inhibitor IV on OVA-induced inflammatory cell infiltration

**Figure 4:**
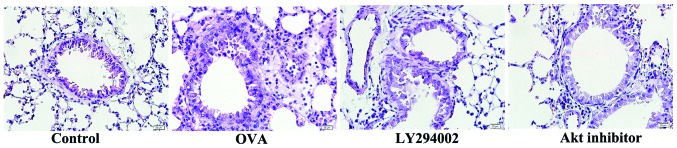
Effects of LY294002 and Akt inhibitor IV on inflammatory cell infiltration. Following administration of LY294002 and Akt inhibitor IV to the asthmatic mouse model, the inflammatory cell infiltration was alleviated (hematoxylin and eosin; magnification, ×400). Akt, protein kinase B.

To determine the role of the PI3K/Akt signaling pathway in inflammatory cell infiltration, LY294002 and Akt inhibitor IV were administered intratracheally to the asthmatic mouse model. Histological analysis, as presented in Fig. 4, demonstrated that LY294002 and Akt inhibitor IV may attenuate inflammatory cell infiltration as compared with the OVA-induced asthmatic group.

### LY294002 and Akt inhibitor IV may overcome airway remodeling

**Figure 5:**
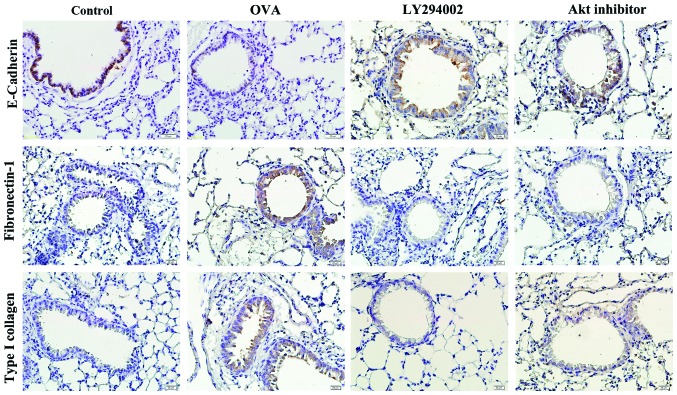
Effects of LY294002 and Akt inhibitor IV on airway remodeling. The expression of E-cadherin was decreased in the OVA-induced mice, while LY294002 and Akt inhibitor IV upregulated the expression. Fibronectin-1 and type I collagen were highly expressed in the epithelial cells of the airways of the OVA-aerosol-challenged mice, and LY294002 and Akt inhibitor reduced their expression (immunohistochemical staining; magnification, ×400). OVA, ovalbumin Akt, protein kinase B.

**Figure 6:**
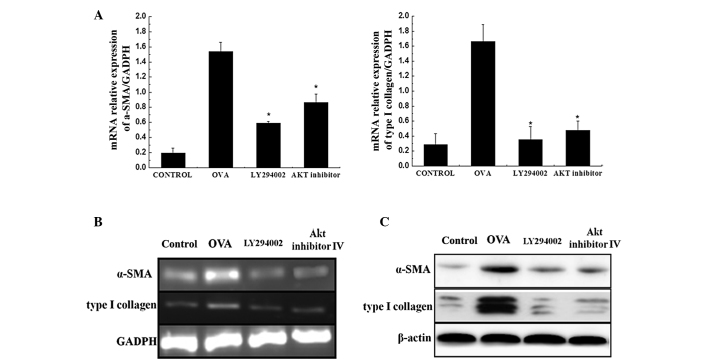
Effects of LY294002 and Akt inhibitor IV on α-SMA and type I collagen. (A and B) Quantitative PCR demonstrated that the mRNA expression levels of α-SMA and type I collagen were higher in the OVA-induced mice compared with the control, and LY294002 and Akt inhibitor IV significantly decreased their expression. (C) The same result may be observed in the protein expression levels as shown by western blot analysis. ^*^P<0.05, vs. control. α-SMA, α smooth muscle actin; OVA, ovalbumin; PCR, polymerase chain reaction; Akt, protein kinase B.

To verify the effects of the PI3K/Akt pathway on OVA-induced airway remodeling, the expression levels of E-cadherin, fibronectin-1 and type I collagen in the airway epithelium cells were detected by immunohistochemistry following LY294002 and Akt inhibitor IV intratracheal administration. High expression levels of fibronectin-1 and type I collagen were identified in the epithelial cells of the airway in the asthmatic mice; LY294002 and Akt inhibitor IV reduced this expression compared with the OVA group. By contrast, the expression level of E-cadherin decreased in the OVA-induced mice, and LY294002 and Akt inhibitor IV were shown to upregulate the expression (Fig. 5). In addition, higher expression levels of α-SMA and type I collagen were identified at the mRNA and protein levels in the OVA-induced mice. Therefore, blocking the PI3K/Akt pathway may inhibit the production of α-SMA and type I collagen (Fig. 6).

## Discussion

In the present study, FIZZ1 was demonstrated to be overexpressed in the OVA-induced asthmatic mouse model and was shown to promote Akt phosphorylation *in vitro*. By blocking the PI3K/Akt pathway, inflammatory cell infiltration in the OVA group was alleviated. In the OVA group, the expression levels of α-SMA, type I collagen and fibronectin-1 were upregulated in the epithelial cells, while the expression of E-cadherin was decreased, indicating that EMT is induced in the airways of asthmatic mice. LY294002 and Akt inhibitor IV can intervene with this EMT process by increasing E-cadherin expression and downregulating the expression of α-SMA, type I collagen and fibronectin-1. Thus, we hypothesize that FIZZ1 may promote airway remodeling in asthmatic mice via the PI3K/Akt signaling pathway, and that blocking the PI3K/Akt pathway may relieve airway remodeling by regulating the abnormal process of EMT in OVA-induced mouse models.

Bronchial asthma is a chronic inflammatory disease of the airways. Airway remodeling, first reported by Huber in 1922 (11), is the main pathological feature of asthma and is the result of sustained inflammation and epithelial cell damage in response to airway injuries (12). The main characteristics of airway remodeling include epithelial detachment, subepithelial fibrosis, airway smooth muscle cell and goblet cell hypertrophy and hyperplasia. All the pathological changes are the major cause of the clinical symptoms and loss of lung function (3,13). Therefore, further studies of airway remodeling may provide a novel treatment strategy for asthma.

Previous studies have demonstrated that EMT promotes airway remodeling (7,14). In the present study, the expression levels of α-SMA, type I collagen and fibronectin-1 were observed to be significantly increased and E-cadherin expression was reduced compared with the saline control, indicating the occurrence of the EMT process.

Preliminary study has revealed that the expression of FIZZ1 was enhanced in the epithelium of the asthmatic rats, which stimulated the expression of α-SMA and type I collagen in fibroblasts (10). However, the mechanism by which FIZZ1 functions in this process remains unknown. In the present study, FIZZ1 was demonstrated to be capable of regulating this process via the PI3K/Akt signaling pathway.

PI3K has a wide range of biological effects in numerous types of immune cells. A previous study revealed that PI3K enzyme activity was increased in OVA-induced murine models of asthma, along with p-Akt, one of the downstream signaling molecules of PI3K (15). LY294002, a specific PI3K inhibitor, is able to significantly downregulate Akt phosphorylation and suppress inflammatory cell infiltration, mucus production and airway hyperresponsiveness in a murine asthmatic model (16). Inflammation and airway remodeling is reduced in PI3Kγ-deficient mice (17). The PI3K/Akt signaling pathway is important in asthma and can promote airway inflammation and hyperresponsiveness, upregulate T-helper 2 cytokine levels and increase mucus production (18). In the present study, following intratracheal administration of LY294002 and Akt inhibitor IV, the infiltration of inflammatory cells was relieved and the markers of EMT, α-SMA, type I collagen and fibronectin-1, expression levels were downregulated with E-cadherin expression increased when compared with the asthmatic mouse model. This result indicates that the PI3K/Akt pathway is involved in the EMT process. Thus, we hypothesize that by blocking the signaling pathway, the process of EMT may be interrupted, which in turn relieves airway remodeling in the mouse asthmatic model.

The PI3K/Akt signaling pathway may be blocked by phosphatase and tensin homolog deleted on chromosome 10 (PTEN) through dephosphorylating the signaling lipid, phosphatidylinositol 3,4,5-triphosphate (19). PTEN protein expression and activity are decreased in allergen-induced asthma (20). Intratracheal administration of adenoviruses carrying PTEN cDNA can reduce the levels of interleukin-4 and −5 in bronchoalveolar lavage fluid, bronchial inflammation and airway hyperresponsiveness (21). A previous study demonstrated that PTEN inhibited the proliferation and migration of human airway smooth muscle mass cells by downregulating the activity of the Akt signaling pathway (22). Numerous studies indicate that PTEN plays a significant role in asthma. However, whether FIZZ1 is an upstream regulator of PTEN, that affects the process of EMT by regulating the PI3K/Akt pathway, remains unknown. Thus, this is the direction of research for future studies.

In summary, the results of the present study demonstrate that FIZZ1 promotes airway remodeling via the PI3K/Akt signaling pathway. In addition, blocking the PI3K/Akt signaling pathway may alleviate airway remodeling in the early stages by intervening with the EMT process. Antagonism of the PI3K/Akt signaling pathway may be a potentially useful strategy in the therapeutic intervention for asthma.

## Figures and Tables

**Table I:** Animal model generation.

Group	Sensitize on days 1 and 14	Challenge on days 21, 22 and 23	Intratracheal agents
Control	Saline	Saline	Saline
OVA	OVA	OVA	Saline
LY294002	OVA	OVA	LY294002
Akt inhibitor IV	OVA	OVA	Akt inhibitor IV

OVA, ovalbumin; Akt, protein kinase B.
